# Age exacerbates the effect of myopia on retinal capillaries and string vessels

**DOI:** 10.3389/fmed.2023.1112396

**Published:** 2023-08-04

**Authors:** Carol Ren Lin, Abduqodir Toychiev, Reynolds Kwame Ablordeppey, Miduturu Srinivas, Alexandra Benavente-Perez

**Affiliations:** Department of Biological Sciences, SUNY College of Optometry, New York, NY, United States

**Keywords:** myopia, string vessels, vasculature, marmoset, branchpoints, retina

## Abstract

The retinal vasculature supplies oxygen and nutrition to the cells and is crucial for an adequate retinal function. In myopia, excessive eye growth is associated with various anatomical changes that can lead to myopia-related complications. However, how myopia-induced ocular growth affects the integrity of the aged retinal microvasculature at the cellular level is not well understood. Here, we studied how aging interacts with myopia-induced alteration of the retinal microvasculature in fourteen marmoset retinas (*Callithrix jacchus*). String vessel and capillary branchpoint were imaged and quantified in all four capillary plexi of the retinal vasculature. As marmosets with lens-induced myopia aged, they developed increasing numbers of string vessels in all four vascular plexi, with increased vessel branchpoints in the parafoveal and peripapillary retina and decreased vessel branchpoints in the peripheral retina. These myopia-induced changes to the retinal microvasculature suggest an adaptive reorganization of the retinal microvascular cellular structure template with aging and during myopia development and progression.

## Introduction

Myopia (nearsightedness) is a refractive error that increases the risk of visual impairment (1–4). It has incurred significant public health implications and is projected to affect 4,758 million people by 2050 (5). Although the increase in myopia prevalence and predicted public health crisis are recognized, the mechanisms that make myopia a significant risk factor for visual impairment remain unknown (1, 2). To date, there are not any available strategies available to prevent myopic degeneration (5).

Myopic eyes experience blur in part due to being larger in size, which can result in compromised vascular support to the inner retina (6). Alterations in the ocular vasculature have been reported in human and experimental models of myopia. Choroidal thinning has been described in human eyes as well as marmosets, mice, and chick models of myopia (7–14). The common marmoset is an established non-human primate (NHP) model that has high predictive value for changes that may occur in human diseases both systemic and ocular (7, 15–19). Both human and primate eyes with no myopic degeneration show larger foveal avascular zones (20), decreased capillary density (5, 21), narrowing of retinal vessel diameters (22), decreased peripheral vessel branching (23), increased parafoveal string vessels (23), and lower central retinal artery blood velocities (22, 24). High myopes with significantly larger eye sizes exhibit decreased ocular perfusion pressure (OPP) as the subfoveal and peripheral choroid thins (13, 25). In marmosets, the OPP is stable during the first year of life but appears to increase with myopia development, possibly related to the changes in metabolic demand that occur as myopic eyes grow larger (26).

Retinal health relies on the interplay between the vasculature, retinal neurons, glial cells, and the extracellular matrix (27). Together, these cells support normal neuronal function and work to provide nutrition (27), metabolic and homeostatic regulation (27–29), and debris phagocytosis (28). During both normal and abnormal development, the blood vessels, glial cells, and ganglion cells work together in a reciprocal feedback loop (30). With the onset of systemic pathology, the neurovascular unit exerts a biphasic influence, experiencing a remodeling reaction that might be harmful in the acute phase and beneficial in the chronic phase (31). Due to the tight relationship between the components of the neurovascular unit, the vascular changes observed in myopic eyes might in turn impair normal vascular and neuronal function, becoming a part of the series of events preceding overt retinal complications associated with myopia (32, 33). However, despite recent progress in the field, the cumulative effects of myopia development and age on the retinal microvasculature cellular structure remain unexplored.

Here, we describe changes to the retinal microvasculature cellular structure in marmosets of different ages with induced myopia. The assessment of the retinal vasculature in all four capillary plexi during myopia development revealed an increase in string-like formation between vascular capillaries and altered blood vessel branching, which are markers observed in vascular pathologies (34–37).

## Methods

### Marmoset model of myopia

Seventeen marmoset eyes were studied: five 6-month-old untreated controls, six 6-month-old myopes, three 12-month-old controls, and three 12-month-old myopes. Both cohorts of myopic eyes were induced with myopia by imposing hyperopic defocus using full-field negative single-vision soft contact lenses (−5D and −10D) (23, 27). The normal lifespan of a common marmoset is 7–8 years in captivity and maximum lifespan of 16–21 years (38–41). In summary, animals initiated treatment at 10 weeks of age, and were treated with either −5D or −10D contact lenses for 16 weeks (6-month-old marmosets), or 42 weeks (12-month-old marmosets). Earlier studies and statistical power analysis of the principle methods used indicated that 3 animals per experimental group provided 80% power for our statistical analysis (*n* = 3 younger control, *n* = 3 younger myope, *n* = 3 older control, *n* = 3 older myope). All animal care, treatment, and experimental protocols were approved by the SUNY College of Optometry Institutional Animal Care and Use Committee (IACUC), the ARVO statement for the use of animals in ophthalmic and vision research, the US National Research Council’s Guide for the Care and Use of Laboratory Animals, the US Public Health Service’s Policy on Humane Care and Use of Laboratory Animals, and the Guide for the Care and Use of Laboratory animals.

At baseline and at end of treatment, cycloplegic refractive error (Rx) and ocular axial length (AL) were measured using the Nidek ARK-700A autorefractor (Nidek Co., LTD, Aichi, Japan) and an ultrasound biometer (Panametrics, NDT Ltd., Waltham, MA, United States) prior to tissue collection under anesthesia (alphaxalone, 15 mg/kg, IM).

### Tissue collection

At the end of treatment, eyes were enucleated and placed in phosphate-buffered saline (PBS; ThermoFisher, Waltham, MA, United States). Dissected retinas were fixed in Para-Formaldehyde (PFA) 4% in PBS (Santa Cruz Biotechnology, Dallas, TX, United States) for 40 min, washed five times for 30 min each with PBS, and incubated with 5% normal goat serum (ThermoFisher) and 0.5% TritonX (Sigma Aldrich, St. Louis, MO, United States) blocking buffer to avoid non-specific antibody binding. Following blocking, the retina was incubated with antibodies diluted in blocking buffer at 4°C for 3 days. The antibody used in this study was isolectin–Alexa 488 (1:100; ThermoFisher). After the incubation period, the retinas were washed five times for 30 min each with PBS. SuperFrost slides (ThermoFisher) were cleaned with ethanol prior to plating. Retinas were inspected for any signs of debris, and consistent tissue thickness achieved by pinching and cutting vitreal remains. Retinas were plated and cover slips were placed on objectives with DAPI mounting medium (Vector Laboratories, Newark, CA, United States), were permitted to self-seal, and stored at −20°C.

### Confocal microscopy and image acquisition

**Figure 1:**
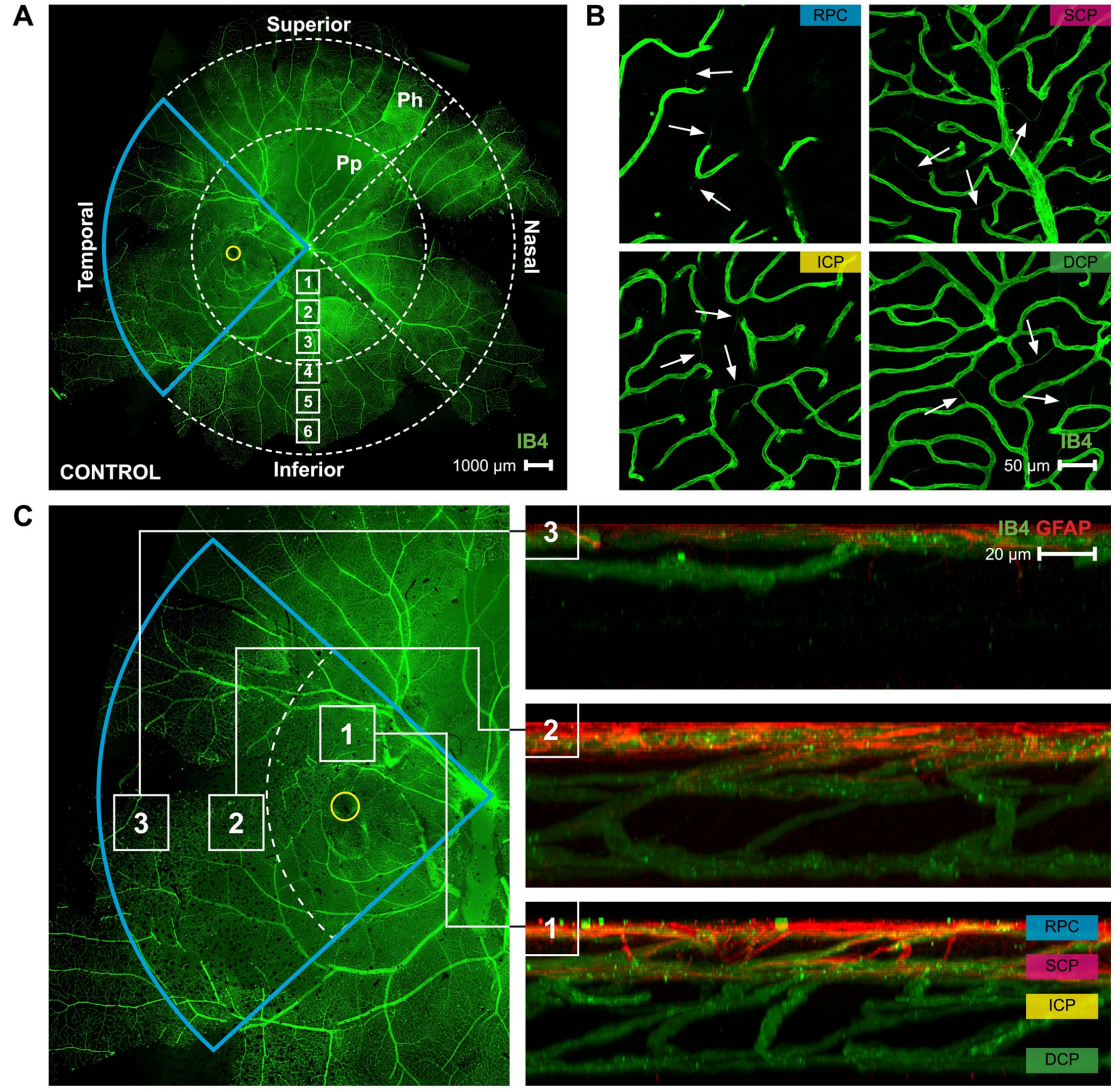
A map of the superficial vasculature of a whole mount marmoset retina, and images of the four different vascular plexi. **(A)** A complete of control marmoset retinal vasculature [green (ID: C16 Left)]. The temporal region of the eye is outlined in blue, and the position of the fovea is indicated by the yellow circle. The retinal vasculature was visualized with conjugated IB4-488. Images were acquired at 4× magnification and stitched in Photoshop. Location of peripapillary region (Pp) and peripheral region (Ph) described in this study is shown. White boxes represent focal areas away from the optic disc to periphery. Boxes labeled “Pp” represent locations where peripapillary location images were taken, while boxes labeled “Ph” represent locations where peripheral location images were taken. Superior, inferior, nasal, and temporal quadrants of the retina are shown. Scale, 1,000 μm. **(B)** Representative images of the retinal vasculature (green) acquired from the parafoveal area which show all vascular layers. Scale, 50 μm. White arrows point to string vessels found in the marmoset retinas. **(C)** The blue insert from 1A is highlighted here, with areas 1, 2, and 3 marked with white boxes indicating the (1) parafoveal region, (2) peripapillary region, and (3) peripheral region. Reconstructed images of areas 1, 2, and 3 can be seen in 1C right, showing the distribution of the retinal vasculature (green) and co-localized astrocytes (red) in those areas. The four vascular plexi are the radial peripapillary capillary (RPC), superficial (SCP), intermediate (ICP), and deep (DCP) plexi. Scale, 20 μm. Figure modified from Lin et al. (23).

The immunohistochemical samples were imaged using the Olympus FV1200 MPE confocal microscope. The images were gathered, and the analyses were performed in a randomized manner by one blind investigator. Sixteen images (317 μm × 317 μm along the horizontal plane, and 10 μm along the vertical plane) were taken from each of the fourteen retinas imaged. Multi-plane z-series were collected using a 40× objective, with each section spaced 1 μm apart. These 10 sections were processed by the confocal microscope to form a single z-stack of images subtending the whole specimen. The number of string vessels per mm^2^ and vessel branch points per mm^2^ were assessed by imaging all four retinal quadrants (temporal, nasal, superior, and inferior) in the periphery, peripapillary, parafoveal, and foveal retina (Figure 1A). After enucleation, right and left eyes were kept separately, and denotation of the temporal region was marked by the presence of the foveal pit (yellow circle, Figure 1A). Nasal is directly opposite of the temporal region, and depending on the eye, superior and inferior retina was categorized. This regional analysis was performed with the goal to identify local changes that might occur in myopic eyes due to their asymmetric eye growth pattern.

### Image and statistical analysis

Blood vessel branchpoints and number of string vessels per mm^2^ were manually counted for each frame on the branches of all orders and converted to number of branch points/mm^2^ and number of string vessels/mm^2^, respectively. The branch points and string vessels were quantified in the radial peripapillary capillary plexus (RPC, most superficial), superficial capillary plexus (SCP), intermediate capillary plexus (ICP), and deep capillary plexus (DCP). Images were processed using Fiji software. A simple geometric correction for magnification along the two-dimensional plane was performed to account for myopic retinal stretch. Data were assessed for normality and analyzed using student t-test, and one-way analysis of variance (ANOVA) and post-hoc analysis using Tukey tests at the level of α = 0.05 were used to examine the differences between treatment and control groups. Pearson’s linear correlation was used to explore the relationship between effective age, axial length, and refractive error and compensatory string vessel and branchpoint measurements. Figures were made using OriginPro 2023 software (OriginLab, Northampton, United States) and assembled in Adobe Indesign (Adobe, San Jose, United States).

## Results

### Retinal vascular plexi in the common marmoset

Marmoset retinas exhibit four vascular plexi in the parafoveal region (Figures 1B,C, area 1): the RPC, SCP, ICP, and DCP. String vessels, thin non-functional connective tissue strands that are remnants of capillaries, are identified in the different plexi of marmoset retinas as white arrows in Figure 1B. In the parafoveal region, there are four vascular plexi [modified from Lin et al. (23); Figure 1C, area 1]. In the peripapillary region, marmosets have three vascular plexi (Figure 1C, area 2): the SCP, ICP, and DCP. The peripheral region (Figure 1C, area 3) only contains two vascular plexi: the SCP and the DCP. In all four groups of marmosets studied, the vasculature by the optic nerve head contained vessels of varying diameters, while the peripheral vasculature appeared to be smaller and uniform in width. Specifically, the average vein vessel width in focal regions 1–3 of the 6-month-old (6 m) marmosets was 18.06 ± 2.9 μm, while the average artery width in the same regions of the 6 m marmosets was 15.74 ± 3.1 μm (*p* > 0.05). The average vein vessel width in focal regions 4–6 of the 6 m marmosets was 12.25 ± 3.4 μm, while the average artery width was 9.95 ± 2.2 μm (*p* > 0.05). Identification, age, axial length, and refractive error of control and myopic marmosets are listed in Table 1.

**Table 1:** Treatment started at 10 weeks of age (72.0 ± 5.5 days) following our established protocol [30–32].

6 m control ID, eye	Age (days)	Gender	Axial length (mm)	Refractive error (D)	6 m myope ID, eye	Age (days)	Gender	Axial length (mm)	Refractive error (D)
C16, right	268	Female	10.259	−0.66	B17, right	214	Female	10.900	−7.93
C16, left	268	Female	10.241	−0.13	B17, left	214	Female	10.894	−7.97
G16, left	215	Male	10.279	−1.15	O17, right	204	Male	10.492	−7.28
H16, right	205	Female	10.286	−0.63	O17, left	204	Male	10.212	−3.91
H16, left	205	Female	10.307	−1.12	P17, right	183	Female	10.554	−7.96
					P17, left	183	Female	10.464	−3.08
AVG ± SD	232.2 ± 32.9		10.27 ± 0.03	−0.74 ± 0.4	AVG ± SD	200.3 ± 14.2		10.61 ± 0.3	−7.01 ± 1.8
	*p* > 0.05		*p* < 0.05	*p* < 0.01					
**12 m control ID, eye**	**Age (days)**	**Gender**	**Axial length (mm)**	**Refractive error (D)**	**12 m myope ID, eye**	**Age (days)**	**Gender**	**Axial length (mm)**	**Refractive error (D)**
X15, right	381	Female	10.216	−1.12	I19, right	388	Male	10.936	−7.34
X15, left	381	Female	10.2202	−1.04	J19, right	388	Male	10.791	−3.48
S15, right	396	Female	10.181	−1.22	J19, left	388	Male	10.766	−3.82
AVG ± SD	386 ± 8.7		10.20 ± 0.02	−1.12 ± 0.1	AVG ± SD	388 ± 0.0		10.83 ± 0.1	−4.08 ± 2.1
	*p* > 0.05		*p* < 0.05	*p* < 0.01					

Lenses were inserted daily in the morning between 8 and 10 a.m. when lights were turned on in the animal room (700 lux) and removed 9 h later at lights off each day (9 h light/15 h dark) (28, 30). Contact lenses had 6.5 mm diameter, 3.6/3.8 mm base curve, were made of methafilcon A (55% water content, DK, 17), fit 0.10 mm flatter than the flattest keratometry measurement, and were assessed using an ophthalmoscope. No corneal complications were observed in any of the animals treated in this or any earlier studies with marmosets (28, 30).

**Figure 2:**
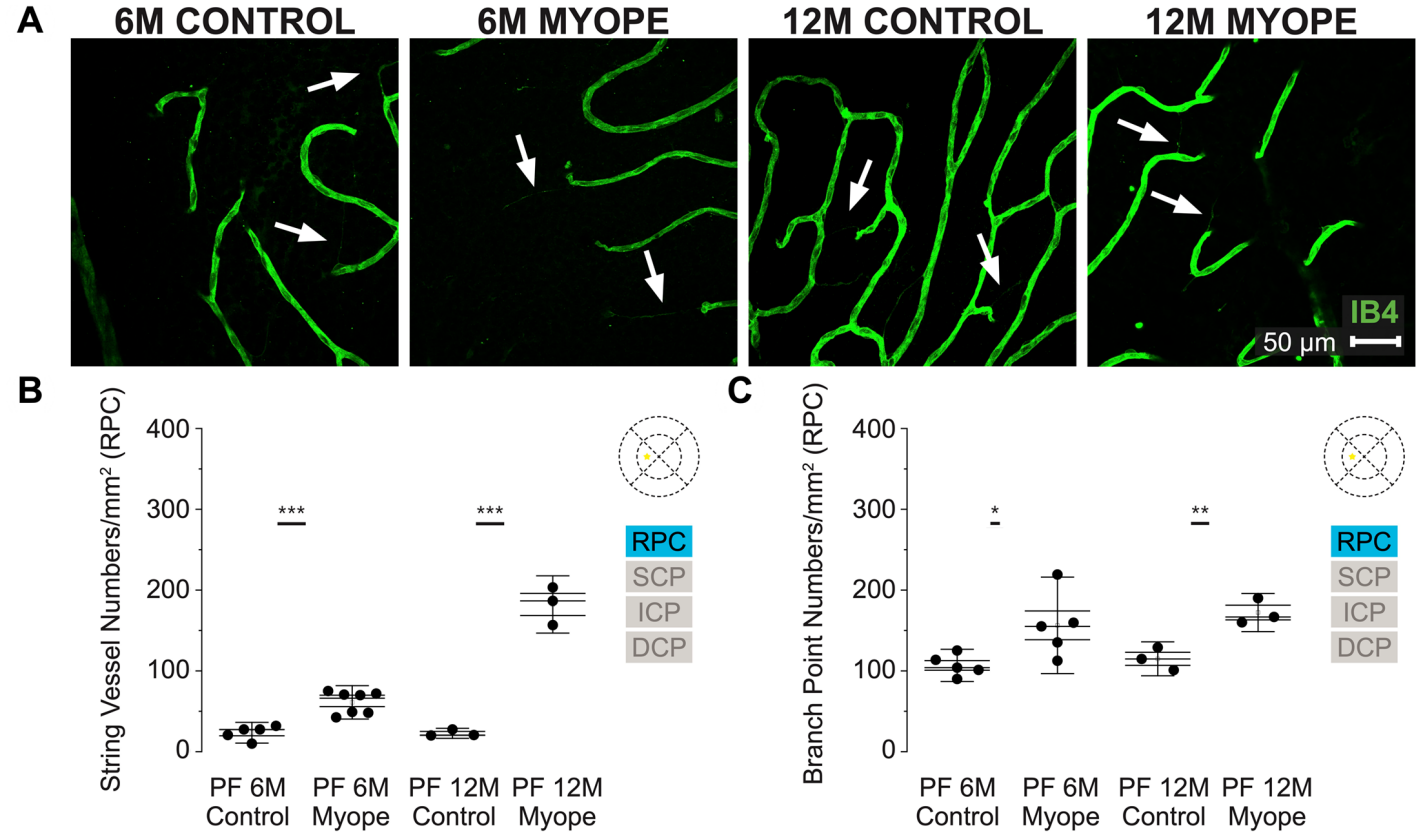
Vascular alterations of the myopic marmoset retina, shown with representative images of the parafoveal RPC plexus in six-month-old (6 m) controls, 6 m myopes, twelve-month-old (12 m) controls, and 12 m myopic marmoset retinas and subsequent analysis of the number of string vessels per mm^2^ and branchpoints per mm^2^ within the RPC of the parafovea. **(A)** Representative images of radial peripapillary capillary plexus vessel structure in the parafoveal region of a 6 m control (ID tag: C16 Right), 6 m myope (ID tag: P17 Right), 12 m control (ID tag: X15 Right) and 12 m myope (ID tag: I19 Right) taken at 40× magnification. Vasculature is labeled with IB4 (green). Scale, 50 μm. White arrows point to string vessels found in the marmoset retinas. ^*^p < 0.05, ^**^p < 0.01, ^***^p < 0.001. **(B)** Analysis of the number of string vessels per mm^2^ in the parafoveal’s RPC layer of the superior, inferior, and nasal retina. Data is shown in as a I-graph box plot for 6 m control (*n* = 5), 6 m myopic (*n* = 5), 12 m control (*n* = 3), and 12 m myopic (*n* = 3) marmoset retinas. Inner box lines represent standard error (SE), and outer lines/whiskers represent standard deviation (SD). A significant increase in string vessels in the 6 m myopic parafoveal RPC was noted (*p* < 0.001) and even more significant increase in the 12 m myopic parafoveal RPC (*p* < 0.001). **(C)** Analysis of the RPC vessel branch points in the parafovea region (6 m control *n* = 5, 6 m myope *n* = 6, 12 m control *n* = 3, 12 m myope *n* = 3). A significant increase in branching was seen in the 6 m myopic parafoveal RPC, with more significant increase noted in the 12 m myopic parafoveal RPC (*p* < 0.05; *p* < 0.01).

**Figure 3:**
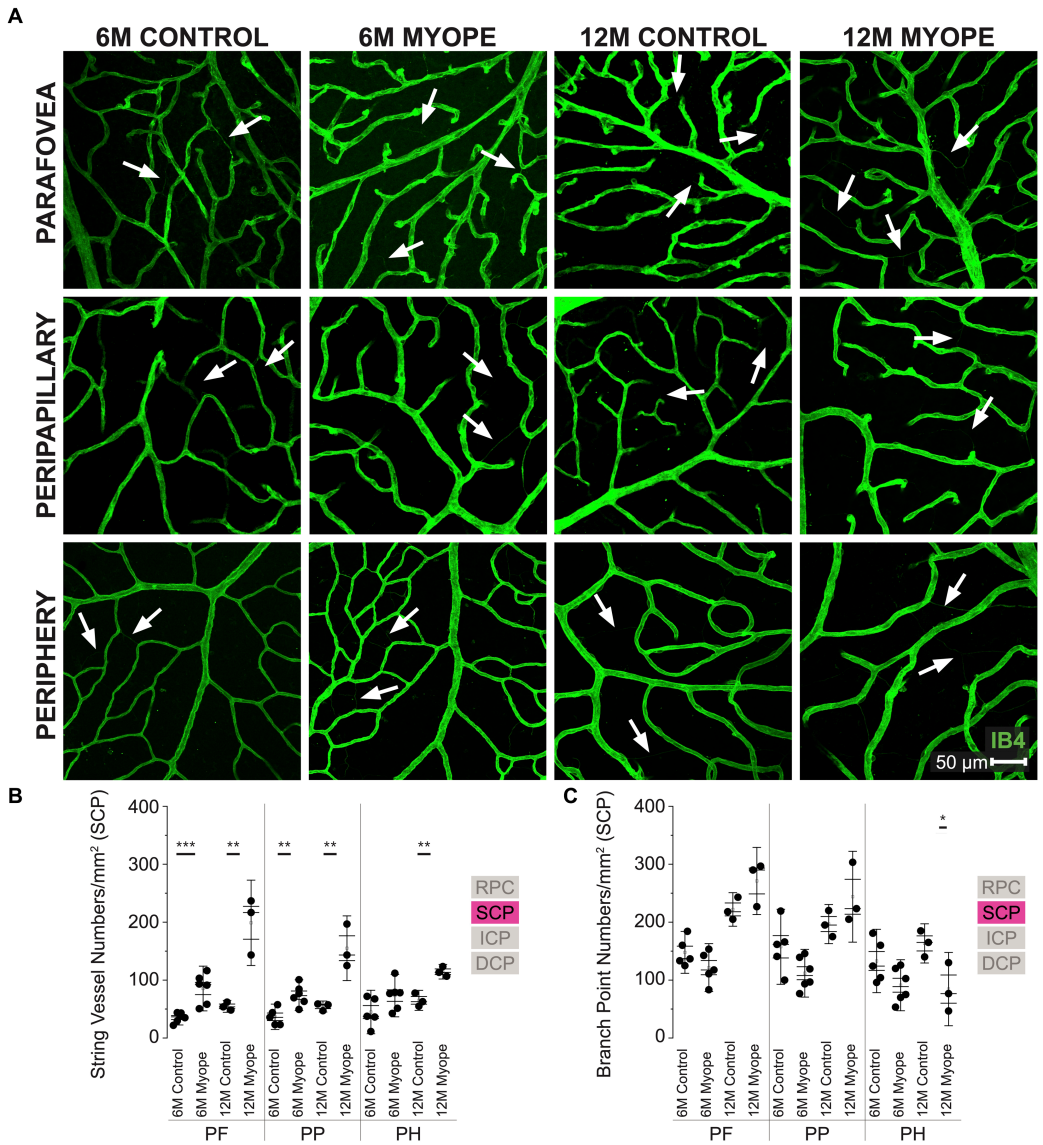
Vascular alterations of the myopic marmoset retina, shown with representative images of pan-retinal SCP plexus in 6 m controls, 6 m myopes, 12 m controls, and 12 m myopic marmoset retinas and subsequent analysis of the number of string vessels per mm^2^ and branchpoints per mm^2^ within the SCP. **(A)** Representative images of superficial capillary plexus vessel structure in the parafoveal, peripapillary, and peripheral region of a 6 m control (ID tag: C16 Right), 6 m myope (ID tag: P17 Right), 12 m control (ID tag: X15 Right), and 12 m myope (ID tag: I19 Right) taken at 40× magnification. Vasculature is labeled with IB4 (green). Scale, 50 μm. White arrows point to string vessels found in the marmoset retinas. ^*^p < 0.05, ^**^p < 0.01, ^***^p < 0.001. **(B)** Analysis of the number of string vessels per mm^2^ in the SCP of the superior, inferior, and nasal retina (6 m control *n* = 5, 6 m myope *n* = 6, 12 m control *n* = 3, 12 m myope *n* = 3). A significant increase in string vessels in the 6 m myopic parafoveal SCP was noted (*p* < 0.001) and an even more significant increase in the 12 m myopic parafoveal SCP (*p* < 0.01). A significant increase in string vessels in the 6 m myopic peripapillary SCP was noted (*p* < 0.01) and an even more significant increase in the 12 m myopic parafoveal SCP (*p* < 0.01). No significant difference was found in string vessels in the 6 m myopic peripheral SCP was noted (*p* = 0.09) with a significant increase in the 12 m myopic peripheral SCP (*p* < 0.01). **(C)** Analysis of the number of vessel branch points in the SCP of the superior, inferior, and nasal retina (6 m control *n* = 5, 6 m myope *n* = 6, 12 m control *n* = 3, 12 m myope *n* = 3). No significant difference was found in SCP branch points of the parafovea or peripapillary myopic eyes, however a significant decrease in peripheral SCP branchpoints per mm^2^ in the 12 m myopic SCP was noted (*p* < 0.05).

**Figure 4:**
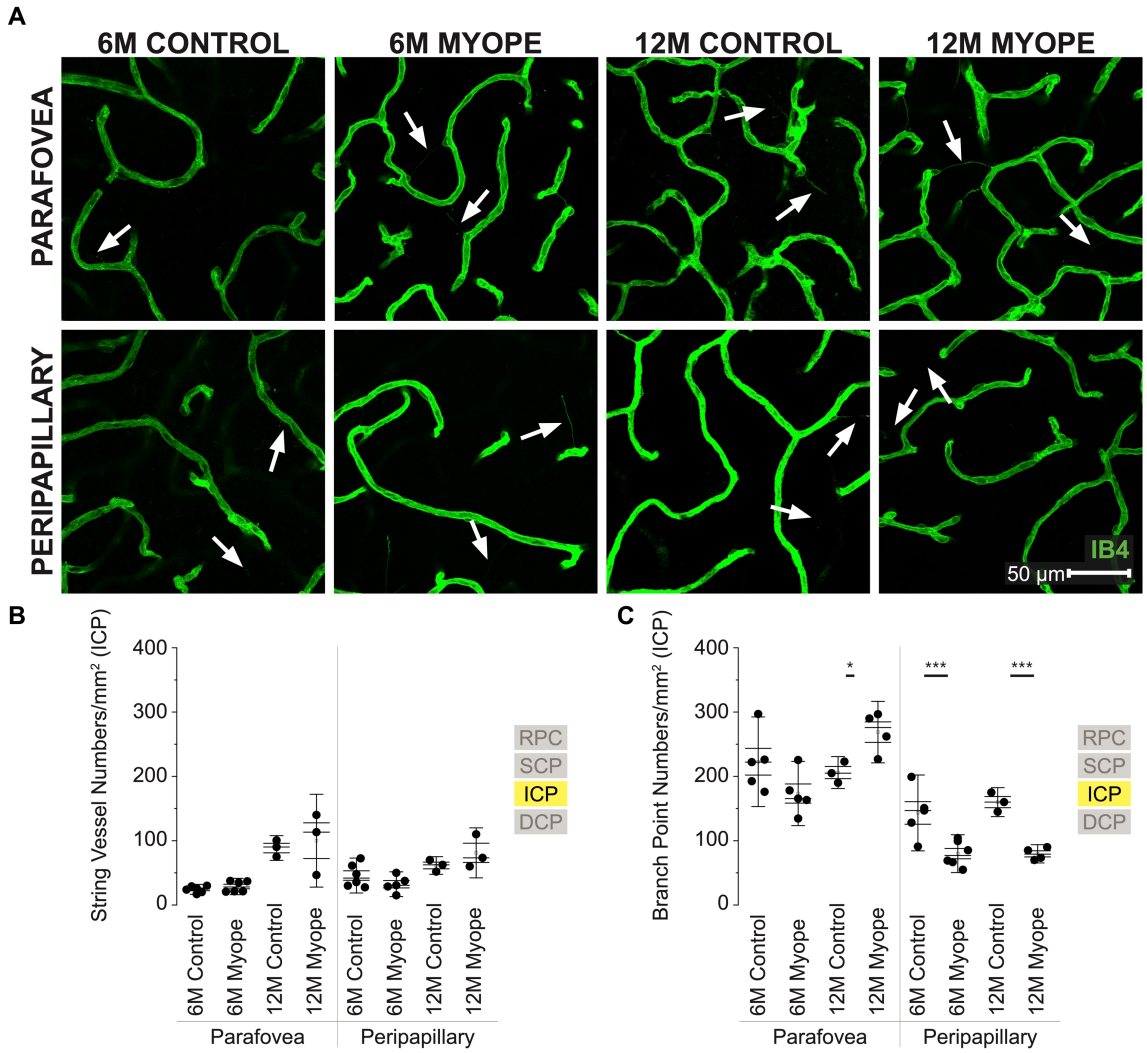
Vascular alterations of the myopic marmoset retina, shown with representative images of parafoveal and peripapillary ICP plexus in 6 m controls, 6 m myopes, 12 m controls, and 12 m myopic marmoset retinas and subsequent analysis of the number of string vessels per mm^2^ and branchpoints per mm^2^ within the ICP. **(A)** Representative images of intermediate capillary plexus vessel structure in the parafoveal and peripapillary region of a 6 m control (ID tag: C16 Right), 6 m myope (ID tag: P17 Right), 12 m control (ID tag: X15 Right), and 12 m myope (ID tag: I19 Right) taken at 60× magnification. Vasculature is labeled with IB4 (green). Scale, 50 μm. White arrows point to string vessels found in the marmoset retinas. ^*^p < 0.05, ^**^p < 0.01, ^***^p < 0.001. **(B)** Analysis of the number of string vessels per mm^2^ in the ICP of the superior, inferior, and nasal retina (6 m control *n* = 5, 6 m myope *n* = 6, 12 m control *n* = 3, 12 m myope *n* = 3). No significant difference in string vessels in the myopic parafoveal or peripapillary ICP was noted (*p* > 0.05). **(C)** Analysis of the number of vessel branchpoints per mm^2^ in the ICP of the superior, inferior, and nasal retina (6 m control *n* = 5, 6 m myope *n* = 6, 12 m control *n* = 3, 12 m myope *n* = 3). A significant increase in parafoveal ICP branchpoints per mm^2^ in the 12 m myopic parafoveal ICP was noted (*p* < 0.05) and significant decrease in parafoveal ICP branchpoints per mm^2^ in the 6 m myope (*p* ≤ 0.01). A significant decrease in peripapillary ICP branchpoints per mm^2^ in the 6 m myopic ICP was noted (*p* < 0.001) as well as in the 12 m myope peripapillary ICP (*p* < 0.001).

**Figure 5:**
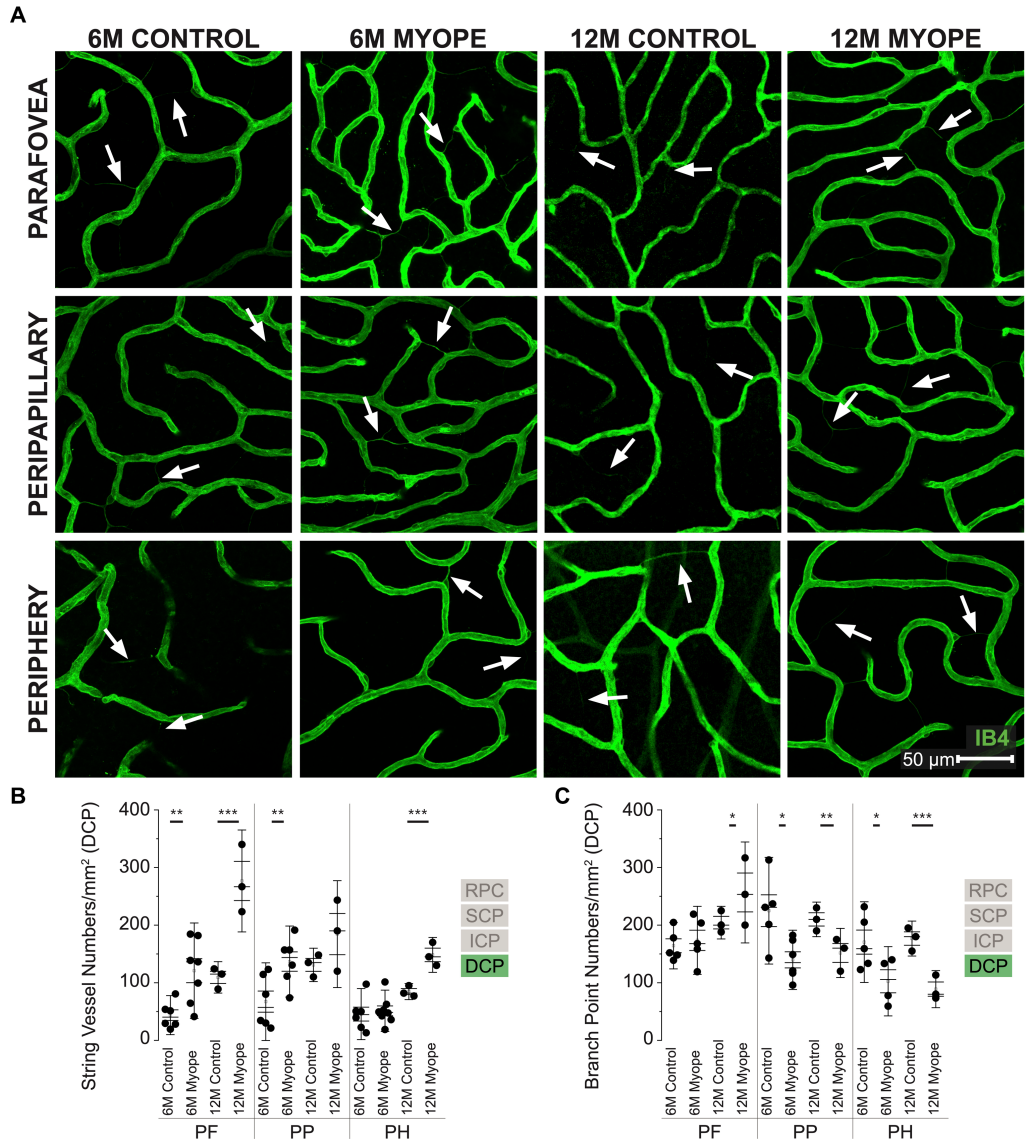
Vascular alterations of the myopic marmoset retina, shown with representative images of pan-retinal DCP plexus in 6 m controls, 6 m myopes, 12 m controls, and 12 m myopic marmoset retinas and subsequent analysis of the number of string vessels per mm^2^ and branchpoints per mm^2^ within the DCP. **(A)** Representative images of deep capillary plexus vessel structure in the parafoveal, peripapillary, and peripheral region of a 6 m control (ID tag: C16 Right), 6 m myope (ID tag: P17 Right), 12 m control (ID tag: X15 Right), and 12 m myope (ID tag: I19 Right) taken at 60× magnification. Vasculature is labeled with IB4 (green). Scale, 50 μm. White arrows point to string vessels found in the marmoset retinas. ^*^p < 0.05, ^**^p < 0.01, ^***^p < 0.001. **(B)** Analysis of the number of string vessels per mm^2^ in the DCP of the superior, inferior, and nasal retina (6 m control n = 5, 6 m myope *n* = 6, 12 m control *n* = 3, 12 m myope *n* = 3). A significant increase in string vessels in the 6 m myopic parafoveal DCP was noted (*p* < 0.01) and even more significant increase in the 12 m myopic parafoveal DCP (*p* < 0.001). A significant increase in string vessels in the 6 m myopic peripapillary DCP was noted (*p* < 0.01) but no difference was noted in the 12 m myopic peripheral DCP (*p* =0.22). No significant difference was noted in string vessels in the 6m myopic peripheral DCP (*p* = 0.69) and a significant increase in 12 m myopic peripheral DCP string vessels per mm^2^ was noted (*p* < 0.001). **(C)** Analysis of the number of vessel branch points in the DCP of the superior, inferior, and nasal retina (6 m control *n* = 5, 6 m myope *n* = 6, 12 m control *n* = 3, 12 m myope *n* = 3). No significant difference in parafoveal DCP branchpoints per mm^2^ in the 6mmyopic parafoveal DCP was noted (*p* = 0.6), with a significant increase in 12 m myopic parafoveal DCP branchpoints per mm^2^ was seen (*p* < 0.05). A significant decrease in both 6 m myopic peripapillary DCP (*p* < 0.05) and 12 m myopic peripapillary DCP branchpoints per mm^2^ (*p* < 0.01) was noted. A significant decrease in peripheral DCP branchpoints per mm^2^ in the 6 m myope (*p* < 0.05) and 12 m myope (*p* < 0.001) was noted.

The older myopic retinas show increased number of string vessels pan-retinally in all four layers of the retinal vasculature compared to younger myopic, younger control, and older control retinas (Figures 2B, 3B, 4B, 5B), as well as increased capillary branchpoint numbers in the central retina, and decreased capillary branchpoint numbers in the periphery (Figures 2C, 3C, 4C, 5C). Capillary branchpoints/mm^2^ was also greater in the innermost vascular layers of the older myopic marmosets, and lower in the outermost vascular layers in all retinal locations (Figures 2C, 3C, 4C, 5C). In the 12-month-old (12 m) marmosets, the average vein and artery vessel widths in focal regions 1–3 were 16.12 ± 1.8 μm and 11.87 ± 2.7 μm, respectively (*p* > 0.05), and 12.04 ± 2.2 μm and 9.49 ± 2.0 μm in focal regions 4–6 (*p* > 0.05). These data suggest that vascular changes are taking place in aging myopic primate retina compared to young myopic retinas.

### Vascular changes in the parafoveal radial peripapillary capillary plexus of the myopic marmosets

The radial peripapillary capillary plexus (RPC) was present in all animals and all quadrants of the parafoveal region imaged (Figure 2A). The RPC is located in the retinal nerve fiber layer, limited to the posterior pole and normally located on the temporal side of the retina. We found that in this plexus 6 m myopic retinas had greater string vessel numbers/mm^2^ compared to controls (Figure 2B; 6 m control 23.5 ± 8.6 string vessels/mm^2^; 6 m myope 61.04 ± 13.8; *p* < 0.001). The 12 m myopic retinas also exhibited greater string vessel numbers/mm^2^ compared to 12 m controls (Figure 2B; 12 m control 22.71 ± 4.2 string vessels/mm^2^; 12 m myopes 182.22 ± 23.6 string vessels/mm^2^; *p* < 0.001). The string vessel density in 6 m controls was not significantly different to that of 12 m controls (*p* > 0.05).

The number of capillary branchpoints/mm^2^ in the RPC was also significantly greater in 6 m myopic retinas compared to 6 m controls, and these differences were greater in the older 12 m myopic retinas compared to 12 m controls (Figure 2C: 6 m control 106.79 ± 13.3 branchpoints/mm^2^, 6 m myope 156.34 ± 39.8, *p* < 0.05; 12 m control 114.91 ± 14.0, 12 m myope 172.22 ± 15.8; *p* < 0.01). Overall the parafoveal RPC of myopic marmosets had greater string vessels and vessel branchpoints numbers than controls. The number of string vessels increased by 150/mm^2^ in 12 m myopic marmosets relative to 12 m control marmosets, and by 40/mm^2^ in 6 m myopic marmosets relative to 6 m control marmosets. The number of branchpoints/mm^2^ increased by 50/mm^2^ in 6 m myopic marmosets relative to 6 m control marmosets, and by 60/mm^2^ in 12 m myopic marmosets relative to 12 m control marmosets.

### Vascular changes in the superficial capillary plexus of the myopic marmosets

The superficial capillary plexus (SCP) was present in all animals, all quadrants and areas imaged (Figure 3A). The string vessels/mm^2^ densities was greater in 6 m myopic marmoset retinas, and even greater in 12 m myopic retinas compared to that of 6 m or 12 control retinas pan-retinally, respectively (Figure 3B: parafovea 6 m control 35.2 ± 8.4 string vessels/mm^2^, 6 m myope 85.42 ± 25.7, *p* = 0.001; 12 m control 54.62 ± 6.9; 12 m myope 198.89 ± 49.1, *p* < 0.01. Peripapillary 6 m control 36.63 ± 14.6 string vessels/mm^2^, peripapillary 6 m myope 73.96 ± 17.7, *p* < 0.01; peripapillary 12 m control 54.36 ± 6.3, 12 m myope 155.0 ± 37.2; *p* < 0.01. Periphery 6 m control 44.88 ± 25.0 string vessels/mm^2^, periphery 6 m myope 73.23 ± 24.4, *p* = 0.09; periphery 12 m control 65.03 ± 11.5, 12 m myope 114.44 ± 8.4, *p* < 0.05).

In this plexus, the number of capillary branchpoints/mm^2^ was unchanged in the 12 and 6 m myopic marmoset retinas compared to 12 and 6 m controls in the parafovea and peripapillary regions (Figure 3C: parafovea 6 m control 147.83 ± 24.1 branchpoints/mm^2^, 6 m myope 121.5 ± 27.6, *p* = 0.14; 12 m control 222 ± 19.3, 12 m myope 271.11 ± 38.6, *p* = 0.12; Peripapillary 6 m control 157.5 ± 43.22 branchpoints/mm^2^, 6 m myope 111.81 ± 27.4, *p* = 0.06; 12 m control 196.6 ± 22.5, 12 m myope 227.22 ± 67.8, *p* = 0.22). There was a significant decrease in capillary branchpoint density in the retinal periphery of 12 m myopic marmoset retinas compared to 12 m controls (Figure 3C right: Periphery 6 m control 133.0 ± 36.4 branchpoints/mm^2^, peripheral 6 m myope 91.11 ± 29.3, *p* = 0.06; peripheral 12 m control 163.33 ± 22.5, 12 m myope 84.44 ± 22.2, *p* < 0.05). The SCP of 12 m myopic marmosets contained more string vessels than 12 m controls, 6 m myopes and 6 m controls did. The SCP vascular branchpoint density was greater in the peripheral retina of 12 m control marmosets. Across the retina, string vessel density increased by 100/mm^2^ in 12 m myopic marmosets relative to 12 m control marmosets, and by 40/mm^2^ in 6 m myopic marmosets relative to 6 m control marmosets. Similarly, capillary branchpoint density decreased in the peripheral retina by 80/mm^2^ in 12 m myopic marmosets relative to 12 m control marmosets, and by 40/mm^2^ in 6 m myopic marmosets relative to 6 m controls.

### Vascular changes in the intermediate capillary plexus of the myopic marmosets

The intermediate capillary plexus (ICP) was present in the peripapillary and parafoveal regions of all animals, in all quadrants evaluated (Figure 4A). The ICP was not present in the periphery. In the parafovea, the number of string vessels/mm^2^ was not significantly different in 6 m or 12 myopic marmoset retinas (Figure 4B: parafovea 6 m control 24.33 ± 5.1 string vessels/mm^2^, 6 m myope 28.75 ± 8.5, *p* = 0.30; 12 m control 88.58 ± 12.8, 12 m myope 100 ± 18.1, *p* = 0.71. Peripapillary 6 m control 45.83 ± 18.1 string vessels/mm^2^, 6 m myope 32.38 ± 2.8, *p* = 0.19; 12 m control 61.46 ± 9.1, 12 m myope 81.11 ± 25.9; *p* = 0.28).

In the parafovea and peripapillary, the capillary branchpoint density was higher in 12 m myopic parafovea retina compared to 12 m controls, but lower in both 6 m and 12 m myopic peripapillary retinas compared to 6 and 12 m controls, respectively (Figure 4C: parafovea 6 m control 222.76 ± 46.4 branchpoints/mm^2^, 6 m myope 173.33 ± 33.3, *p* = 0.09; 12 m control 206.0 ± 16.5, 12 m myope 271.11 ± 38.6, *p* < 0.05. Peripapillary 6 m control 143.18 ± 39.3 branchpoints/mm^2^, 6 m myope 79.86 ± 19.6, *p* < 0.01; 12 m control 160.0 ± 15.0, 12 m myope 77.78 ± 10.7; *p* < 0.001).

Overall the ICP in 12 m myopic marmosets contained similar amounts of string vessels/mm^2^ than 12 m controls, 6 m controls or 6 m myopes across the retina. ICP vascular branchpoints increased in the parafoveal retina of 12 m myopic marmosets and decreased in the peripapillary retina of 6 and 12 m myopic marmosets. The number of branchpoints/mm^2^ increased by 70/mm^2^ in the parafovea of 12 m myopic marmosets relative to 12 m control marmosets, and decreased by 50/mm^2^ in peripapillary of 6 m myopic marmosets relative to 6 m controls. Similarly, the number of branchpoints per mm^2^ in the parafovea decreased by 80/mm^2^ in 12 m myopic marmosets relative to 12 m control marmosets.

### Vascular changes in the deep capillary plexus of the myopic marmosets

The DCP was present in all animals and all quadrants imaged (Figure 5A). In the parafovea and peripapillary, the number of string vessels/mm^2^ was greater in 6 m myopic marmoset retinas compared to 6 m controls, and even greater in 12 m myopic retinas compared to 12 m control retinas (Figure 5B: parafovea 6 m control 43.75 ± 22.6 string vessels/mm^2^, 6 m myope 120.89 ± 55.2, *p* < 0.01; parafovea 12 m control 109.33 ± 18.2, 12 m myope 276.67 ± 59.0, *p* < 0.01. Peripapillary 6 m control 67.19 ± 44.9 string vessels/mm^2^, 6 m myope 136.77 ± 41.1, *p* = 0.01; peripapillary 12 m control 131 ± 19.3, 12 m myope 184.44 ± 61.9, *p* = 0.22). The number of string vessels/mm^2^ was greater in the periphery of 12 m myopic retinas compared to 12 m controls (Periphery 6 m control 45.52 ± 29.5 string vessels/mm^2^, 6 m myope 51.11 ± 25.9, *p* = 0.69; 12 m control 84.67 ± 9.3, 12 m myope 148.3 ± 20.2; *p* < 0.01).

The number of parafoveal DCP vessel branchpoints/mm^2^ in 6 m and 12 m myopes was not different from that of 6 or 12 m controls, respectively (Figure 5C: parafovea 6 m control 164.26 ± 26.8 branchpoints/mm^2^, 6 m myope 173.57 ± 39.4, *p* = 0.67; 12 m control 204.33 ± 18.9, 12 m myope 256.67 ± 58.4, *p* = 0.21). In the peripapillary and peripheral retina, the number of capillary branchpoints/mm^2^ was lower in 6 m and 12 myopic retinas compared to that of 6 m control or 12 m control retinas, respectively (Figure 5C: peripapillary 6 m control 225.0 ± 61.8 branchpoints/mm^2^, 6 m myope 139.76 ± 34.3, *p* = 0.01; peripapillary 12 m control 210.0 ± 20.0, 12 m myope 151.67 ± 28.4, *p* < 0.05. Periphery 6 m control 170.51 ± 46.7 branchpoints/mm^2^, 6 m myope 102.55 ± 40.1, *p* < 0.05; periphery 12 m control 176.67 ± 20.27, 12 m myope 88.89 ± 21.4; *p* < 0.01).

The DCP of 12 m myopic marmosets contain had greater string vessel density than 12 m controls, 6 m controls or 6 m myopes pan-retinally. DCP vascular branchpoints/mm^2^ decreased in the peripapillary and periphery retina of 12 m myopic marmosets. Across the retina, the number of string vessels per mm^2^ increased by 50/mm^2^ in 12 m myopic marmosets relative to 6 m myopic marmosets, and by 100/mm^2^ relative to 12 m control marmosets. Similarly, the number of branchpoints per mm^2^ across the retina decreased by 60/mm^2^ in 12 m myopic marmosets relative to 6 m myopic marmosets, and by 110/mm^2^ relative to 12 m control marmosets.

### Regression analysis

Stepwise multiple regression models were used to evaluate whether the numbers of string vessel/and branchpoints observed would be predicted by the age, axial length, or refractive error of the animals. In these models, age, axial length, or refractive error were the independent variables, and the string vessel and branchpoint measures in each ETDRS region and layer were the dependent variables. As myopic eyes aged, they grew longer and had relatively higher numbers of string vessels and decreased branchpoints in the SCP (multiple regression, R^2^ = 0.16, *p* < 0.001), ICP (multiple regression: R^2^ = 0.272, *p* < 0.001) and DCP (multiple regression; R^2^ = 0.321, *p* < 0.001). Decreasing branchpoints/mm^2^ of the SCP were negatively correlated with increasing axial length (R^2^ = 0.83, *p* < 0.001) and higher refractive error (R^2^ = 0.69, *p* < 0.001).

## Discussion

This study provides evidence of significant changes in all four capillary plexi of the retinal vasculature in marmosets induced with myopia, a NHP model successfully used in vision research due to the similarities in structure and function to the human eye. Compared to 6-month myopic and 6-month old controls, 12-month old myopic marmosets had greater numbers of string vessels in all capillary plexi, and increased branchpoint density in the parafoveal and peripapillary retina. The confocal images obtained confirm the presence of four vascular plexi in the marmoset retina, and the presence of string vessels, similar to human retinas (35–37, 42). These plexi, from inner to outer retina, are the radial peripapillary capillaries (RPC), superficial capillary plexus (SCP), intermediate capillary plexus (ICP), and deep capillary plexus (DCP) (43).

The retina is one of the most energy demanding tissues in the body (44, 45). Several studies suggest that microvasculature changes can be markers of neurological and ocular diseases (35, 46–51), contribute to abnormal blood flow changes (24, 32, 52–54), and compromise vascular integrity resulting in reduced metabolic support (27, 51, 55, 56). In this study, vascular remodeling and plasticity was observed in marmoset retinas with induced myopia, and the changes observed were exacerbated by age. The marmoset (*Callithrix jacchus*) has been established as an excellent non-human primate model in vision and neuroscience research due to its fast development, small size, diurnal foveated retinas, ease in breeding and handling, and high optical quality eye (7, 16, 19). NHP are critically important for the development of human treatments (15, 17, 18).

Age-related conditions such as Alzheimer’s disease, dementia and hypertension have been associated with changes in ocular microvasculature (35–37, 51, 57, 58). In this study, retinal microvasculature alterations were observed in marmosets induced with myopia. Specifically, an increase in string vessels and branchpoint densities in all capillary plexi at the parafovea and peripapillary retina were observed as myopic marmosets aged. These findings are in line with others seen in the human retina (35–37); string vessels are present in diabetic retinopathy before microvascular changes occur (34), making the identification of string vessels a vital part of vascular disease management. The RPC is considered highly vulnerable to insult and damage due to high metabolic demand of retinal ganglion cells (RGC) (59–61). There is evidence that structural changes to the RPCs are associated with the pathogenesis of age-related RGC axonal loss in humans (62). RPC loss and glaucomatous nerve fiber layer damage has also been identified in patients with chronic glaucoma (63). The increased string vessel and branchpoint density observed in the parafoveal RPC of marmosets induced with myopia suggests that the myopic retinal microvasculature might be experiencing capillary regression and string vessel formation, similarly to that described in several vascular diseases (51, 64–66). There is also evidence of an increase in string vessel formation as a consequence of ganglion cell injury (67), and related to an induced apoptotic phenomenon associated with endothelial cell destruction, attached by macrophages (6).

String vessels are thin connective tissue strands, non-functional remnants of capillaries that do not carry blood flow (36). The presence of string vessels suggests an originally normal-functioning vessel that gradually disappeared after abrupt or chronic ischemia (68), diabetes (34), aging (35), or neurodegenerative disorders (37), among others. While capillary branchpoint density was significantly greater in the RCP of myopic eyes, its density in the SCP, ICP and DCP layers was lower compared to controls. Reduced vessel branching has been associated with decreased retinal blood supply in mice eyes (46, 69), and an aberrant blood vessel development results in decreased density and branching of the capillary network (70). After three weeks of sustained whole-body hypoxia in ten-week-old mice, increased blood vessel branchpoint density have also been shown to be significantly increased (68).

Despite the branchpoint reduction observed in 6 m marmosets, 12 m myopic marmosets showed an increase in retinal string vessels and decrease in peripheral branchpoints. Increased string vessel formation and decreasing number of microvasculature branches have been noted in the normal aging of human cerebral white matter (42, 71–75), and are likely to occur even in the absence of significant neuron loss (37, 71, 73, 76). In the cortical vasculature of 76–81 years old humans with no vascular dementia, there is a decline in capillary surface area and density compared to those of a younger population under 50 years old (77, 78). This indicates the presence of age-related pathology in the cortical microvasculature, that possibly precedes the pathophysiology of vascular disease. Certain microvascular abnormalities may occur prior to development of disease (76), The retina is part of the blood–brain-barrier, and exhibits similar pathological processes as does the cerebrum. Retinal ischemia has been shown to increase the number of string vessels (79), with decreased vascular density in aging animals (33, 70) shown as well.

We also observed an increase in retinal branchpoint density in all four parafoveal vascular layers, and decrease in retinal branchpoint density of the same layers with increasing distance from the optic nerve head, in the 12 m myopic marmosets compared to the values of 6 m myopic marmosets. This is in line with other studies in primate research. Four different capillary networks (the RPC, SCP, ICP, and DCP) with distinct vascular patterns can be found in the control primate and non-primate retina (80), specifically in the macula and peripapillary region (81). In humans, the SCP is highest in the macular regions, and decreases in the periphery (82). The peak vessel density of normal, control SCP of humans is higher than the ICP and DCP in the parafoveal region as well, with the density of the ICP and DCP higher in the periphery than in the SCP (82). All retinal vascular layers in the human eye are densest in the macular/peripapillary region, and reduces in thickness and density with increasing eccentricity (43, 59, 83). During the disease onset of diabetes in human eyes, the density of the SCP, ICP, and DCP progressively decreases toward the periphery, increasing disease severity, and with ganglion cell density decrease (84). In a model of human retinal vascular occlusion, decreased vascular density has been shown in both the superficial and deep vascular networks (85).

In our study, increased age, refractive error, and axial length were associated with increased string vessel density across the SCP, ICP, and DCP retinal vascular layers, and decreased SCP branchpoint density in myopic marmosets. Age appears to exacerbate the effect of myopia on the retinal vasculature, specifically by increasing string vessel density and decreasing branchpoint density with increasing retinal eccentricity. The retinas of older myopes appear to be compensating for increased duration of stress to the vasculature due to the sustained effect of increased myopic growth on the vasculature, and the results shown are in line with previous studies. One study in human myopes showed decreased SCP and DCP vessel densities with age and decreased SCP density with high myopia and longer axial length (22). Another study showed decreased deep vascular plexus density in a human model of high myopia was most associated with high myopia (86). In pathological myopia, alterations to inner retinal microvascular density occur, specifically a decrease in the DCP (87).

## Conclusion

The results from this study indicate that aging exacerbates the effects of myopic eye growth on the architectural template of the retinal vasculature at the cellular level, in all four vascular plexi in a NHP model of lens-induced myopia. The restructuring and reorganization found reflects what might be an adaptation to the sustained mechanical stress of myopic eye growth. Collectively, this may be a part of a beneficial adaptive chronic response to maintain the adequate functioning of retinal neurons during ocular growth (31). Alternatively, the changes to the vasculature seen could represent the opposite, a detrimental response indicating the onset of compromised structural and functional support to the retinal neurons that preserve vision (31). The vascular changes seen in this study may precede pathological myopic changes to the retina like myopic neovascularization, further emphasizing the importance of this work.

Our study confirms the feasibility of the marmoset in studying the retinal vasculature. The aim of the study was to evaluate how aging interacts with the effect of myopic eye growth on the structure and distribution of the retinal vasculature. The findings of this study confirm that myopic eyes without pathology exhibit changes to the numbers of string vessels and branchpoint in all four vascular plexi, suggesting that the vasculature is indeed affected by the mechanical stretch induced by myopia. Whether these changes noted are beneficial or harmful, and whether their function diminishes with disease progression, remains to be seen. Future studies will aim to evaluate quantitatively the functional changes to the vasculature with increasing myopia and increasing age.

## Data availability statement

The raw data supporting the conclusions of this article will be made available by the authors, without undue reservation.

## Ethics statement

The animal study was reviewed and approved by IACUC Ethics Committee of SUNY College of Optometry.

## Author contributions

CL and AB-P contributed to the conception and design of the study. CL, AT, RA, MS, and AB-P contributed to methodology and data curation. AB-P acquired resources and funding for this study. CL performed the statistical analysis and wrote the original draft of the manuscript. All authors contributed to the article and approved the submitted version.

## Funding

This work was supported by the American Academy of Optometry Career Development Award to ABenavente, National Institute of Health’s National Eye Institute T35 grant to CL.

## Conflict of interest

The authors declare that the research was conducted in the absence of any commercial or financial relationships that could be construed as a potential conflict of interest.

## Publisher’s note

All claims expressed in this article are solely those of the authors and do not necessarily represent those of their affiliated organizations, or those of the publisher, the editors and the reviewers. Any product that may be evaluated in this article, or claim that may be made by its manufacturer, is not guaranteed or endorsed by the publisher.
